# Novel *EPHB4* Receptor Tyrosine Kinase Mutations and Kinomic Pathway Analysis in Lung Cancer

**DOI:** 10.1038/srep10641

**Published:** 2015-06-15

**Authors:** Benjamin D. Ferguson, Yi-Hung Carol Tan, Rajani S. Kanteti, Ren Liu, Matthew J. Gayed, Everett E. Vokes, Mark K. Ferguson, A. John Iafrate, Parkash S. Gill, Ravi Salgia

**Affiliations:** 1 Department of Surgery, University of Chicago, Chicago, Illinois, United States of America; 2 Department of Medicine, Section of Hematology/Oncology, University of Chicago, Chicago, Illinois, United States of America; 3 Comprehensive Cancer Center, University of Chicago, Chicago, Illinois, United States of America; 4 Department of Medicine, Division of Medical Oncology, University of Southern California, Los Angeles, California, United States of America; 5 Department of Pathology, Massachusetts General Hospital, Boston, Massachusetts, United States of America

## Abstract

Lung cancer outcomes remain poor despite the identification of several potential therapeutic targets. The EPHB4 receptor tyrosine kinase (RTK) has recently emerged as an oncogenic factor in many cancers, including lung cancer. Mutations of *EPHB4* in lung cancers have previously been identified, though their significance remains unknown. Here, we report the identification of novel *EPHB4* mutations that lead to putative structural alterations as well as increased cellular proliferation and motility. We also conducted a bioinformatic analysis of these mutations to demonstrate that they are mutually exclusive from other common RTK variants in lung cancer, that they correspond to analogous sites of other RTKs’ variations in cancers, and that they are predicted to be oncogenic based on biochemical, evolutionary, and domain-function constraints. Finally, we show that *EPHB4* mutations can induce broad changes in the kinome signature of lung cancer cells. Taken together, these data illuminate the role of EPHB4 in lung cancer and further identify EPHB4 as a potentially important therapeutic target.

Receptor tyrosine kinases (RTKs) are frequently altered in lung cancer. EGFR, MET, RON, KIT, and EPH family members are commonly overexpressed or mutated, contributing to tumorigenesis in the lung. Recently, several members of the EPH family of RTKs have been found to play important roles in lung cancer. Notably, a mutation in *EPHA2* causes constitutive kinase activation in and contributes to the development of lung squamous cell carcinoma (SCC)^1^, while mutations in *EPHB6* appear to have significant pro-metastatic effects in non-small cell lung cancer (NSCLC) cells^2^. *EPHA3* mutations in lung cancer appear to have pro-tumorigenic effects via suppression of the normal function of wild-type EPHA3 as a tumor suppressor in the lung^3^. *EPHA3* and *EPHA5* are frequently altered in NSCLC, though the functional significance of these alterations is unknown^4^. Cross-talk between Akt and EPHB3 has also been proposed in the progression of NSCLC^5^.

EPHB4 is overexpressed and amplified in several lung cancer subtypes and is necessary for the growth of lung adenocarcinoma xenografts in mice^6^. This appears to be mediated by Akt and Src signaling downstream. Though this oncogenic role for EPHB4 in lung cancer has been established, its exact function and signaling partners have not been fully investigated. For example, downstream mediators of EPHB4 activity remain largely unexplored and represent a major area of possible therapeutic potential.

Non-synonymous mutations in the *EPHB4* gene have been identified, and many occur in human tumor tissues and cell lines. For instance, a mutation resulting in an R564K substitution occurring in the intracellular JM domain was detected in one multiple myeloma cell line^7^, and an R889W substitution was detected in one gastric carcinoma tissue sample^8^. Several somatic non-synonymous mutations have also been identified in other EPH receptors and potentially contribute to receptor activation^9^. However, the functional and structural effects of *EPHB4* mutations and their potential significance in the context of lung cancer remain essentially unknown.

Here, we report novel mutations in the *EPHB4* gene that cause putative alterations in protein structure as well as increased proliferation in lung cancer cells, and a bioinformatic analysis of several mutations strengthens their association with lung cancer. The downstream signaling patterns of wild-type and mutated *EPHB4* in lung cancer are also reported using high-throughput kinome signatures.

## Methods

### Tissue procurement

Human lung cancer patient tissues were obtained from the University of Chicago Tumor Tissue Bank. These experiments were approved by the University of Chicago IRB. Tumor tissues were documented along with patient characteristics where available with written informed consent and in accordance with IRB protocol.

### Mutational analysis

Thirty-two lung adenocarcinoma, 46 small cell lung cancer (SCLC), 32 squamous cell lung carcinoma (SCC), 22 squamous cell carcinoma of the head and neck (HNSCC), and 32 pleural mesothelioma tissues were sequenced and analyzed for the presence of *EPHB4* mutations. The majority of specimens were fixed in formalin and embedded with paraffin for long-term storage; total genomic DNA was later extracted using standard procedures for use in mutational analysis. DNA from a panel of cell lines (NSCLC: A549, H226, H358, H522, H661, H1703, H1993, SW1573; SCLC: H69, H82, H249, H345, H2171; normal lung: BEAS-2B; non-lung: 3T3, PC3) was also extracted and used in mutational analysis. Seventeen intronic primer pairs flanking *EPHB4* exons were designed for PCR amplification and are listed in Supplementary Table 1. All primers were designed using Primer3 and purchased from Integrated DNA Technologies (Coralville IA). PCR was performed using Phusion High-Fidelity DNA Polymerase (Finnzymes, Woburn MA) in recommended reaction conditions and using the following touchdown PCR cycling parameters: initial denaturation at 98 °C for 30 s; 10 cycles of denaturation at 98 °C for 5 s, annealing at 73-n°C for 15 s (where n=cycle number), and extension at 72 °C for 15 s; 20 cycles of denaturation at 98 °C for 5 s, annealing at 62 °C for 15 s, and extension at 72 °C for 15 s; and final extension at 72 °C for 5 m. PCR products were run on 1% w/v agarose gels at 100 V for 30 m to confirm the presence of expected band sizes and sequenced at the University of Chicago DNA Sequencing Core Facility. Sequences were analyzed against wild-type *EPHB4* for variations using Sequencher (Gene Codes, Ann Arbor MI) and were further validated using Mutation Surveyor (Softgenetics, State College PA). Variations were discarded if they were not detected in both forward and reverse sequencing reactions and if they were not reproducible upon subsequent sequencing.

### SNaPshot sequencing

Genomic DNA was extracted from formalin-fixed, paraffin-embedded tumor tissues as described above. The SNaPshot mutation detection platform is based on multiplex single-base-extension PCR followed by capillary electrophoresis sequencing using a ABI PRISM 3730 DNA analyzer (Life Technologies/Applied Biosystems, Carlsbad CA) and has been fully established for use in testing clinical samples for the presence of cancer-associated mutations^10^,^11^.

### Bioinformatic analyses and structural modeling

CanPredict^12^,^13^ was used to predict whether a non-synonymous variation would be deleterious to the structure and function of EPHB4 using biochemical, evolutionary, and domain-function constraints^14^,^15^,^16^. mCluster^17^ was used to consolidate and visualize analogous sites of *EPHB4* mutations within other TKD-containing proteins and determine whether mutations have been detected at these sites. PyMOL software (Schrödinger, Portland OR) was used to visualize mutation sites within the EPHB4 protein structure. PSIPRED^18^ was used to predict protein secondary structure from changes to the primary sequence. All gene/protein sequences were acquired from the Ensembl and NCBI databases.

### Cell culture

The NCI-H661 NSCLC cell line was purchased from ATCC and maintained at 37 °C and 5% CO2 in RPMI medium supplemented with 10% fetal bovine serum, 1% penicillin/streptomycin, 2% sodium bicarbonate, 1% sodium pyruvate, 1% HEPES buffer, and 1% L-glutamine.

### Mutagenesis

A wild-type *EPHB4* cDNA clone in the pCMV6-XL6 vector (Origene, Rockville MD) was used as a mammalian expression vector and as a template within which to generate mutants. The QuikChange II Site-Directed Mutagenesis Kit (Stratagene, La Jolla CA) was used to generate isolated single amino-acid changes within the *EPHB4* ORF (G723S, A742V, P881S). Mutation-specific primers are listed in Supplementary Table 1. All mutant constructs were sequenced in forward and reverse directions to confirm successful mutagenesis reactions.

### *Expression of* EPHB4 *constructs in cultured cells*

The wild-type and G723S, A742V, and P881S mutant constructs above were individually transiently transfected into H661 cells or 293T cells. Cells were plated in antibiotic-free medium in either 96-well plates at a density of 2.0 × 10^4^ cells per well (for cell viability assays; eight replicates per experiment) or 10-cm dishes at a density of 3.0 × 10^6^ cells per plate (for lysate collection or cell-based assays) and allowed to grow to approximately 50–75% confluence (to allow for exponential growth over the following 72 hours). Cells were transfected using Lipofectamine 2000 transfection reagent (Invitrogen) according to its standard protocol. Complexes were removed after 4–6 h and replaced with fresh antibiotic-free medium after washing with PBS. Untransfected and mock-transfected (transfection reagent only) cells served as controls. Protein expression was confirmed in H661 cells by immunoblotting (Supplementary Fig. 1), and cellular localization was confirmed in 293T cells by immunofluorescence (Supplementary Fig. 2).

### Cell proliferation assays

Following transfection, cells were left to grow until the desired time point, at which time the media was removed, cells were washed once with PBS, and 100 μL fresh growth medium was added to each well. For ligand stimulation and drug assays, cells were treated with ephrin-B2-Fc (1 μg/mL) followed by paclitaxel (0.5 μM), soluble EPHB4 (sEPHB4, 20 μg/mL), paclitaxel plus sEPHB4, or DMSO as a control. Following the addition of 5 μL of a 0.028% resazurin sodium salt solution (w/v; Sigma, St. Louis MO), plates were incubated at 37 °C protected from light for 2–5 h and fluorescence was measured using a plate reader (530/590 nm ex/em).

### Cell motility assays

Cell motility was determined using a wound healing assay with stably transfected H661 cells. Cells were grown as above to confluence in six-well dishes. At the 0 h time point, linear scratch wounds were made in the monolayers with sterile pipette tips, and movement into the wounds was assessed every 4 hours for a total of 12 hours using an Olympus digital camera with a microscope adapter.

### Ephrin-B2-binding properties and phosphorylation of EPHB4 mutants

A cell-based ephrin-B2-binding assay was performed to assess native receptor-ligand interactions of wild-type and mutant EPHB4. EPHB4-expressing 293T cells were harvested by scraping and incubated with ephrin-B2-AP in PBS for 30 m. Cells were pelleted, the supernatant was discarded, and the pellet was resuspended. Bound ephrin-B2-AP was detected by addition of PNPP (alkaline phosphatase substrate) and measured on a plate reader as optical density.

To assess phosphorylation of EPHB4 mutants, immunoprecipitation was performed. Lysates of 293T cells expressing wild-type or mutant EPHB4 were extracted with Triton X-100, immunoprecipitated with beads linked to anti-EPHB4 antibody^19^ (#47, generously provided by the Gill Laboratory, University of Southern California), and immunoblotted with anti-EPHB4 primary antibody^19^ (#265, generously provided by the Gill Laboratory, University of Southern California) or anti-phosphorylated tyrosine primary antibody (pTyr, #4G10; Millipore, Billerica MA) followed by appropriate secondary antibodies. Band intensities were quantified using an Odyssey chemiluminescence detector.

### PamChip protein tyrosine kinase arrays and reagents

All reagents and PamChip protein tyrosine kinase arrays used in PamGene runs were purchased from PamGene International B.V. (`s-Hertogenbosch, The Netherlands). The PamStation 12 system was also purchased from PamGene^20^.

### Lysate collection

Cells were transfected with 100 nM *EPHB4*-directed siRNA as previously described^6^ or with plasmids and left to grow until the desired time point. Whole-cell lysates were collected as previously described^6^ using M-PER mammalian protein extraction reagent (Pierce, Rockford IL) supplemented with 1.8X protease inhibitor (Pierce) and 1.8X Halt phosphatase inhibitor (Pierce). Protein concentration was estimated using a Nanodrop spectrophotometer (Thermo Scientific, Wilmington DE).

### Peptide phosphorylation assays

For each array, 30 μg whole-cell lysate was added to make a final mixture of 1X protein kinase (PK) buffer, 10 mM dithiothreitol, 1x BSA, 400 μM ATP, FITC-conjugated anti-phosphotyrosine antibody (PY20), and water to total 40 μL. Following a blocking step using 2% BSA and a subsequent wash using 1X PK buffer, this reaction mixture was loaded onto a protein tyrosine kinase PamChip and a run was started and followed the standard PamGene protein tyrosine kinase workflow protocol. Each sample was measure in quadruplicate.

### Run analyses

Raw run data consisting of sample annotations and image files were compiled and analyzed in a semi-automated fashion using the BioNavigator4 software suite provided by PamGene. Peptides were automatically located, identified, and gridded based on an array layout text file containing peptide identities and locations, and the intensity of each spot, which corresponds to a unique peptide substrate, was quantified and integrated for every image to control for image saturation at longer exposure times and increase the dynamic range of detection. Spot intensities from post-wash 100 ms exposure time images were used for further analysis. Intensities were first normalized to the local background signal by subtracting the median background signal from the median spot intensity. The 1st percentile of the resulting spot intensities was calculated, data below this value were cut off (to remove lowest-intensity and very negative outliers whose backgrounds had a stronger intensity than the spots themselves, as this may suggest non-specific antibody binding), and the remaining data were shifted to eliminate values less than 1.0. Data were then log-transformed to normalize the distribution of intensities.

### Statistical analyses

For cell proliferation assays, replicate data points were averaged and compared to time-matched mock-transfected cells. To assess variability between a set of treatment conditions with multiple time points, two-way ANOVA was used. Error bars represent standard error of the mean normalized to percent difference versus control values. All statistical calculations were performed using Prism software (GraphPad, La Jolla CA).

For peptide phosphorylation assays, log intensity values were analyzed using paired two-tailed student’s t tests comparing control samples versus treated samples. Fold changes were calculated by subtracting mean log control values from mean log treatment values. Heat maps were generated using the R statistical package and the RColorBrewer library. Color scale ranges were set arbitrarily based on the highest-amplitude peptide score that met statistical significance within a given run. Peptides that did not meet statistical significance were considered to be unchanged and were therefore assigned a fold change of zero.

### Pathway analyses

Peptides found to be significantly different between *EPHB4* knockdown and control in the statistical analysis (p < 0.05) were used for pathway analysis using the GeneGo pathway analysis package (Thomson Reuters, St. Joseph MI). The top 15 most significant process networks were identified, and relevant signaling networks were assembled based on manually curated objects generated by PamGene fold-change data.

## Results

### Identification of novel mutations of EPHB4

**Figure 1:**
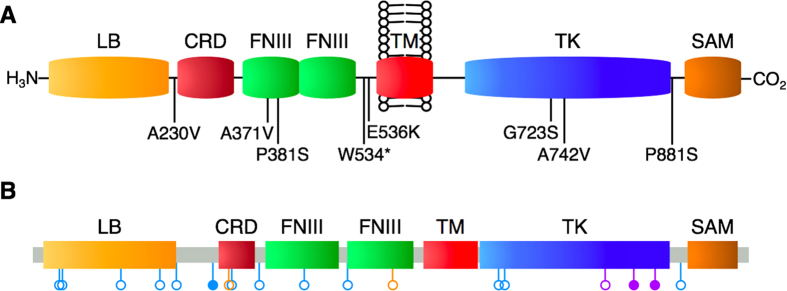
EPHB4 mutations detected in human lung cancer tissues. A schematic of non-synonymous mutation sites within the domain structure of EPHB4 is shown. **A**: *EPHB4* mutations reported in the present study. **B**: Compiled *EPHB4* mutations across all lung cancer datasets currently included in cBioPortal. Blue, adenocarcinoma; purple, squamous cell carcinoma; orange, small cell lung carcinoma. Open circles, non-synonymous point mutations; closed circles, splice site variant or nonsense mutation.

The 17 exons of *EPHB4* were sequenced in lung cancer patient tissues and lung cancer cell lines. No non-synonymous (NS) mutations were detected in cell lines; however, a number of synonymous and NS variations were found in lung tissues (summarized in Fig. 1 and Supplementary Table 2; chromatograms for each are provided in Supplementary Fig. 3; a full list of variations is provided in Supplementary Table 3.) Notably, eight NS *EPHB4* mutations were detected: one (A230V) in an extracellular linker region; two (A371V and P381S) in the first extracellular fibronectin III repeat; two (W534* and E536K) in the extracellular juxtamembrane domain; two (G723S and A742V) in the tyrosine kinase domain; and one (P881S) in an intracellular linker region just carboxy-terminal to the tyrosine kinase domain. Three of these (A230V, A371V, and P381S) occurred in adenocarcinoma, one (A742V) occurred in SCC, and four (W534*, E536K, G723S, and P881S) occurred in SCLC. Seven of these eight mutations (all except A371V) have not been previously detected. Of note, no non-synonymous *EPHB4* mutations were detected in HNSCC or pleural mesothelioma tissues.

### Bioinformatic analyses of mutations

Three of the eight mutations (P381S, G723S, and A742V) were predicted by CanPredict to be associated with cancer, with an additional mutation, P881S, demonstrating an equally low SIFT score (0.00; Table 1). Another encodes an opal stop codon (W534 > stop), and three others are predicted to be benign (A230V, A371V, and E536K); the latter of these was detected in the same patient as the aforementioned stop codon and thus is not expressed.

The two *EPHB4* kinase-domain mutations detected here have also been described at corresponding residues in other kinase family members (Table 2), as detected using mCluster. Specifically, the G723S mutation has been detected in three other kinases and the A742V variant in seven others; several of these analogous sites of variations were detected in malignancies, including lung cancer.

**Figure 2:**
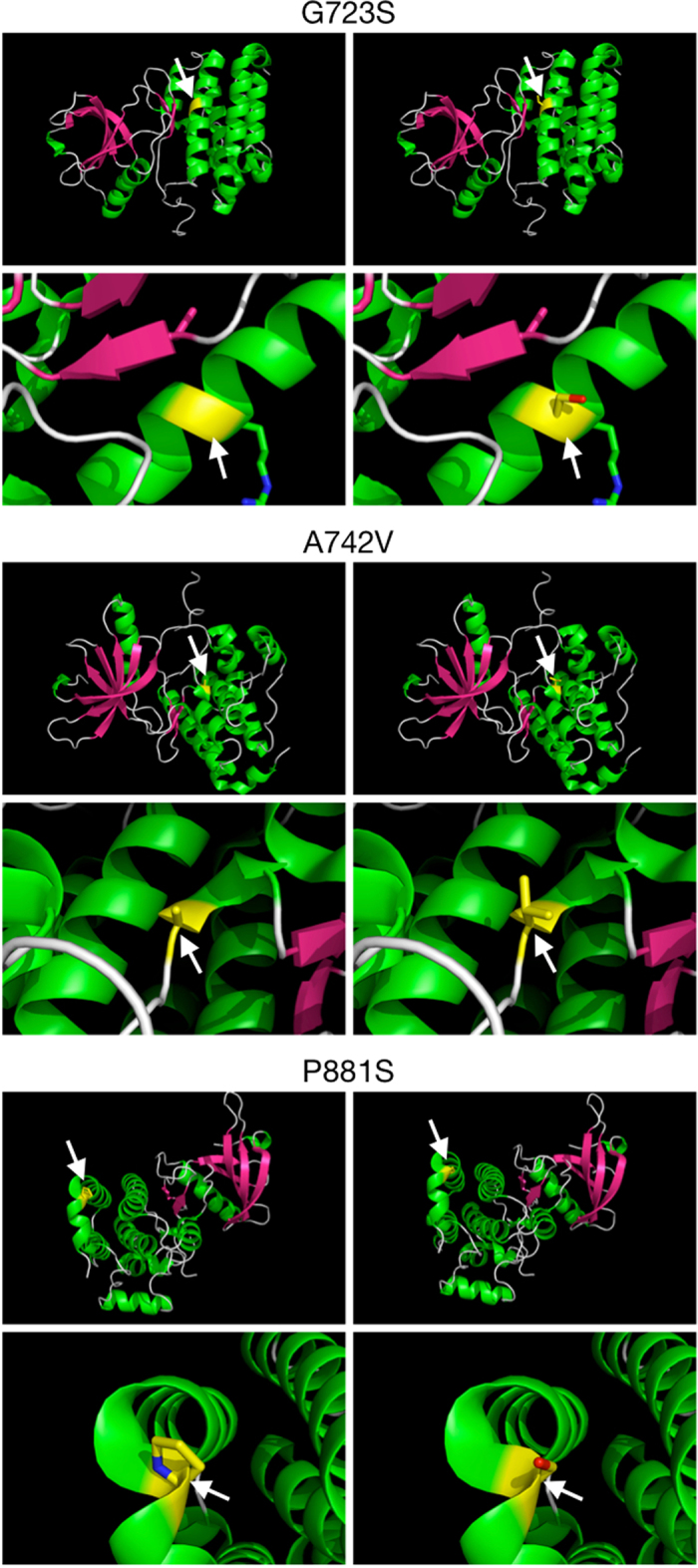
Three-dimensional structures of three non-synonymous EPHB4 mutations detected in lung tumor tissues. For each, wild-type protein is shown in the left panel, and the mutated protein is shown in the right panel. Arrows indicate the residues of interest. All images were created with PyMOL using a crystal structure encompassing the majority of the EPHB4 TK domain (PDB 2VWY; Reference 62). Top: Glycine replaced by serine within an alpha-helix. Middle: Alanine replaced by valine between a turn and linker region. Bottom: Proline replaced by serine within a helix-turn-helix motif.

Three-dimensional structures of the EPHB4 tyrosine kinase domain were generated in order to visualize the potential structural changes that could result from the G723S, A742V, and P881S variations. While the A742V mutation does not appear to have any significant novel interactions with nearby residues, and an alanine-to-valine shift is not significant based on amino acid side chains, G723S and P881S do appear to be more interesting in that they both produce potential serine phosphorylation sites. In particular, the P881S mutation is significant in that prolines commonly induce turns in protein secondary structure, so replacing P881 with serine may also cause more significant changes in protein folding (Fig. 2). Based on a prediction using PSIPRED, the A742V mutation breaks the adjacent L741 residue from the short helix structure involving R739, D740, and L741 in the wild-type protein. Predictions for G723S and P881S revealed that the local helix and coil, respectively, that contain them are likely unaffected by these mutations.

### Mutations in EPHB4 are frequently mutually exclusive from other mutations in proteins commonly aberrant in lung cancer

**Figure 3:**
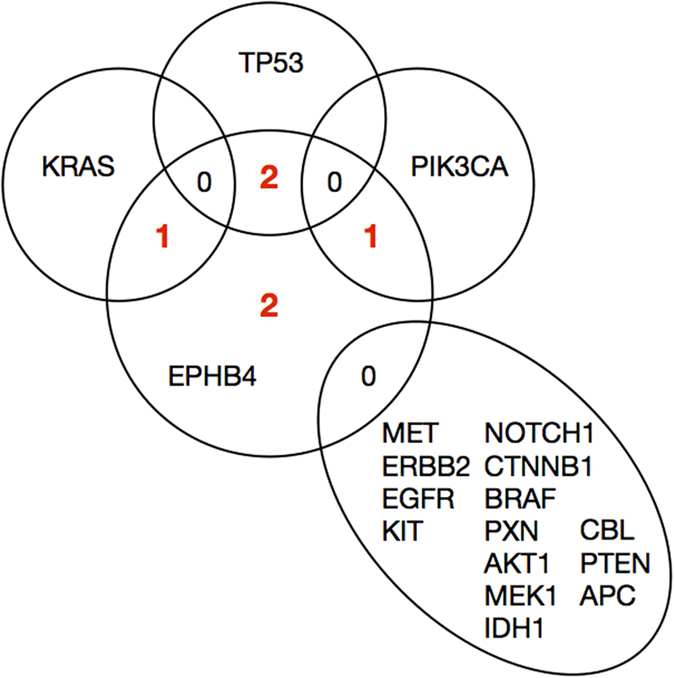
Venn diagram demonstrating mutual exclusivity of EPHB4 mutations with respect to other frequently aberrant proteins in cancer. Of six *EPHB4* mutation-harboring tissues, one also harbored only a G12C mutation in *KRAS*, one only a H1047R mutation in *PIK3CA*, one only a R248Q mutation in *TP53*, and another only a G245C mutation in *TP53* among the common variants investigated; two *EPHB4* mutation-harboring tissues were entirely free from other mutations at the sites investigated. None of the tissues harboring *EPHB4* mutations were found to have variations in the genes indicated in the lower right segment.

Genomic DNA from specimens in which non-synonymous mutations were detected was also sequenced using the SNaPshot platform^11^ at a number of oncogenic loci within other cancer-associated genes. Additionally, all exons of *MET*, *CBL*, and *EGFR* were sequenced in these specimens. Overall, the degree to which non-synonymous *EPHB4* single nucleotide variations were mutually exclusive from others is notable (Supplementary Table 4). Of the 17 additional genes sequenced, non-synonymous mutations were only found in three genes [*KRAS* (G12C), *PIK3CA* (H1047R), and *TP53* (G245C and R248Q)] across four tissues. The vast majority of other loci sequenced were found to be wild-type in all six mutation-harboring tumor tissues (Fig. 3; Supplementary Table 4).

### EPHB4 mutants localize to the cellular membrane and bind ephrin-B2 but are variably phosphorylated

Expression of wild-type and mutant EPHB4 in 293T cells was localized to the cellular membrane (Supplementary Fig. 2). The extent of ephrin-B2 binding was similar among wild-type and mutant EPHB4 (Supplementary Fig. 4A). The ratio of phosphorylated EPHB4 to total EPHB4 among wild-type EPHB4 and the P381S and G723S variants was similar; however, phosphorylation of the A742V and P881S variants was sharply reduced in comparison (Supplementary Fig. 4B).

### Exogenous expression of mutant EPHB4 increases cell proliferation and motility in vitro

**Figure 4:**
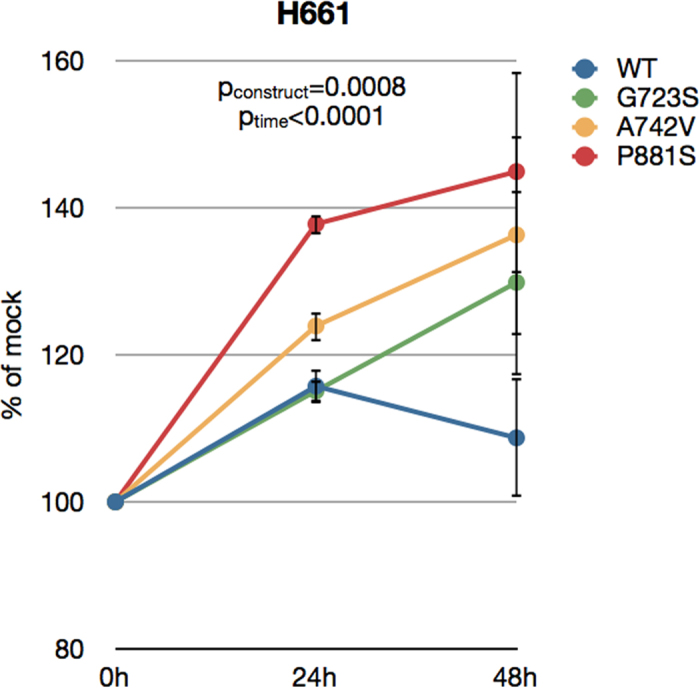
Increased cell proliferation in cells transfected with wild-type or mutant EPHB4. H661 cells were transfected with either wild-type or mutant *EPHB4* constructs, and their effect on cell proliferation was measured over the time points shown using resazurin fluorescence. Untransfected and mock-transfected (transfection reagent only) cells served as controls, and data points represent the percent versus matched mock-transfected cell values at each time point. Each condition was repeated in eight replicates. Error bars indicate SEM. Overall p values shown were calculated by two-way ANOVA for time and construct.

The H661 cell line, which demonstrated very low EPHB4 expression (Supplementary Fig. 1), was used as a model in which to test the effects of exogenous expression of wild-type EPHB4 and mutant forms of EPHB4. We have previously shown that expression of wild-type EPHB4 in H661 cells results in increased proliferation as well as enhanced motility^6^. Based on the earlier bioinformatic analysis of mutations detected here in lung cancer, the G723S, A742V, and P881S mutations were selected for further study. Plasmids containing wild-type *EPHB4* or one of the three mutant forms of *EPHB4* were individually transfected into H661 and their effects on cell viability and motility were observed. Expression of wild-type EPHB4 in the absence of stimulation with ephrin-B2 resulted in a 16% increase in cell proliferation after 24 hours compared to mock-transfected cells, although this gain of function was reduced to a 9% increase the next day. However, after 48 hours, the three mutants tested caused significant increases in cell proliferation. The G723S, A742V, and P881S mutants resulted in 30%, 36%, and 45% increases in proliferation, respectively, over mock-transfected cells after 48 hours (Fig. 4).

**Figure 5:**
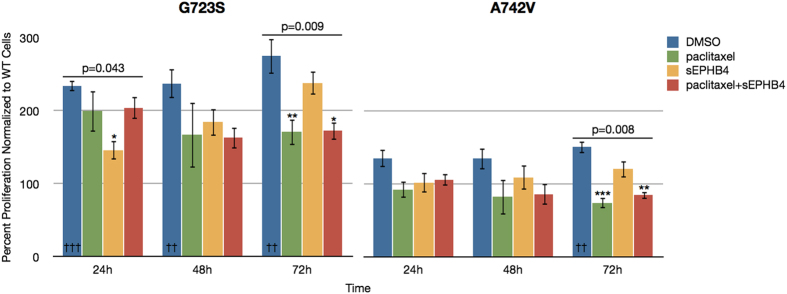
Cell proliferation and drug treatment in cells transfected with mutant EPHB4 in the presence of ephrin-B2 stimulation. H661 cells transfected with EPHB4-G723S or EPHB4-A742V mutant constructs were exposed to ephrin-B2-Fc (1 μg/mL) and treated with DMSO (control), paclitaxel (0.5 μM), soluble EPHB4 (sEPHB4; 20 μg/mL), or paclitaxel plus sEPHB4, and the effects on cell proliferation were measured as previously described. Each condition was repeated in three replicates. Data are expressed as values normalized to EPHB4-WT cells at the same time point and experimental conditions. Error bars indicate SEM. Overall p values shown were calculated by one-way ANOVA for drug treatment at a single time point. Within single time points, * denotes p < 0.05, ** denotes p < 0.01, and *** denotes p < 0.001. †† denotes p < 0.01 and ††† denotes p < 0.001 in comparing each mutant construct without drug treatment to WT cells at the corresponding time point.

We also tested the effects of ephrin-B2 stimulation on cell proliferation alone and in the presence of targeted agents. When stimulated with ephrin-B2 ligand, cells expressing EPHB4 harboring the G723S or A742V mutations exhibited significantly greater proliferation compared to cells expressing wild-type EPHB4. Treatment of cells harboring EPHB4 mutations with paclitaxel and paclitaxel combined with sEPHB4 resulted in significant decreases in cell proliferation; however, in the case of the G723S mutation, these drugs did not completely attenuate the effect of the mutant construct suggesting that this mutation confers some resistance to these agents. Additionally, the inhibitory effect of sEPHB4 became less pronounced with progressive time points, raising the possibility that the effect of the G723S mutation becomes less and less dependent on ligand stimulation. In general, the proliferative effects of the G723S mutation were stronger than those of the A742V mutation (Fig. 5). These data suggest that while wild-type EPHB4 provides some stimulation for cell proliferation, the intracellular mutations detected here provide a significant gain of function with respect to proliferation.

Additionally, H661 cells expressing the A742V mutation in the absence of stimulation with ephrin-B2 were more motile and closed wounds to a greater extent than those expressing wild-type EPHB4 (Supplementary Fig. 5).

### EPHB4 has broad effects on phosphorylation events in lung cancer cells

**Figure 6:**
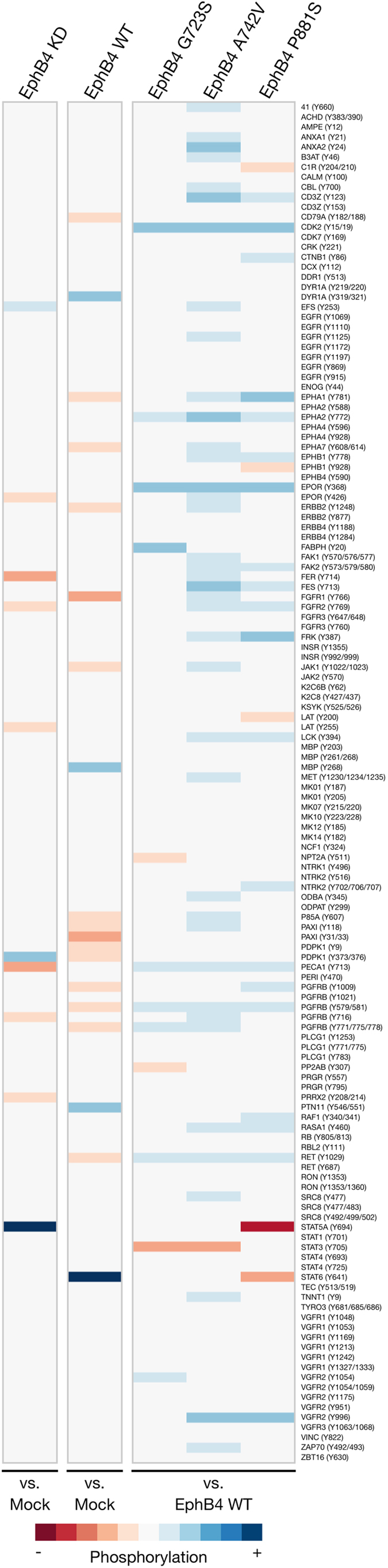
Broad changes in tyrosine kinase phosphorylation after EPHB4 modulation. Heat maps demonstrate fold differences in peptide substrate phosphorylation using the PamGene platform. Peptides interrogated are listed to the right. Red tones indicate decreased phosphorylation and blue tones indicate increased phosphorylation compared to controls indicated below the heat map, and neutral color indicates either slight changes in phosphorylation or changes in phosphorylation that did not meet statistical significance (p < 0.05) across replicates. KD and WT samples were assayed in quadruplicate; mutant samples were assayed in triplicate.

Using the PamGene platform to assay cells in which EPHB4 was knocked down, wild-type EPHB4 was re-expressed, or mutant EPHB4 variants were expressed, it was demonstrated that normal EPHB4 signaling has significant interactions with a vast array of proteins and pathways (Fig. 6).

Notable effects include a mutant-induced increased phosphorylation of CDK2, EPHA2, EpoR, and VEGFR2; decreased EPHA1, PDGFRß, and Ret phosphorylation with wild-type EPHB4 expression but increased phosphorylation with mutant EPHB4 expression; and decreased FGFR2 and PECAM1/CD31 phosphorylation with EPHB4 knockdown and increased phosphorylation with mutant EPHB4 expression. Also notable were a decrease in paxillin phosphorylation following re-expression of wild-type EPHB4, and increased PDPK1 phosphorylation with EPHB4 knockdown and decreased phosphorylation with wild-type EPHB4 expression. Finally, the STAT family was strongly affected by EPHB4 modulation. STAT5A had significantly increased phosphorylation following EPHB4 knockdown and significantly decreased phosphorylation with expression of the P881S EPHB4 variant. STAT3 had a significant decrease in phosphorylation with expression of the G723S and A742V EPHB4 variants. STAT6 also showed a significant decrease in phosphorylation following P881S expression but a significant increase with wild-type EPHB4 expression. Generally, the three EPHB4 mutations tested here, especially the A742V variant, appear to have strong effects toward the broad promotion of tyrosine phosphorylation (Fig. 6). Phosphorylation of several representative targets was validated by immunoblotting (Supplementary Fig. 6).

**Figure 7:**
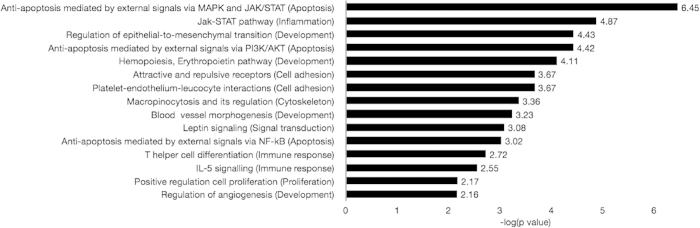
Enriched GeneGo process networks following EPHB4 knockdown. The top 15 most significantly enriched process networks are sorted by statistical significance based on manually curated objects generated by PamGene fold-change data. Data represent the negative log of the p value determined by GeneGo (more significant processes appear nearer the top).

A GeneGo analysis was conducted using data generated with EPHB4 knockdown samples in order to better visualize and interpret these findings. The most active cellular processes include anti-apoptotic, inflammatory, and developmental signaling, many of which involve STATs (Fig. 7). The significantly altered peptides in response to EPHB4 knockdown were also manually curated into a signaling network to demonstrate any signaling interactions between them, and it was found that PECAM1, EpoR, Fer, Lat, and STAT5A interact to some extent with each other (Supplementary Fig. 7). The signaling activity of STAT5A was explored more specifically within the anti-apoptotic process network previously identified, which clearly establishes it as a downstream target of the JAK family of kinases, ERK1/2, and PDGFR and as a regulator of Fos, XIAP, Bcl-XL, Bcl-2, Pim-1, and BFL1 (Supplementary Fig. 8).

## Discussion

We have reported a series of novel non-synonymous mutations in the EPHB4 receptor tyrosine kinase with associated putative structural alterations and effects on kinome signaling. Overall, we found that non-synonymous mutations occurred in 7% of samples overall, with non-synonymous mutation rates of 9% in adenocarcinoma, 9% in SCLC, and 3% in SCC. The A371V variant identified here in lung adenocarcinoma has previously been reported^8^.

A number of *EPHB4* mutations have previously been identified in solid and hematogenous tumor specimens^21^,^22^. Non-synonymous mutations of *EPHB4* were identified in 2% of sequenced lung squamous cell carcinomas^23^, each of which was located in its kinase domain. Another study specifically investigating lung adenocarcinomas reported an *EPHB4* somatic mutation rate of 1-3% of sequenced tumors^24^,^25^. Many others in various other types of tumors and cell lines have been identified and catalogued as part of The Cancer Genome Atlas project^26^; however, few have been studied or characterized in detail, so it is unknown whether they have transforming effects on the EPHB4 protein. Of 16 sites with *EPHB4* mutations in adenocarcinoma tissues, 12.5% were located in the kinase domain.

A similar report was recently published by Mäki-Nevala *et al*. identifying mutations of EPH family members in NSCLC^27^. Several alterations in *EPHA* and *EPHB* receptors were found, and many of these occurred in the kinase domains of their respective proteins. However, their cohort of tissues was limited to NSCLC, and the functional significance of these mutations remains unknown. Furthermore, no mutations in *EPHB4* were identified, and, in contrast to our findings, many of the alterations in EPH receptors co-occurred with other driver mutations in lung cancer. That the data presented here disagree so starkly with these findings is likely due to the diverse cohort of specimens we have interrogated as well as potentially the unique patient characteristics in our American patient population versus those in Finland, who may have a different profile of risk factors, such as unique environmental exposures, variable smoking history^28^, and gender distribution of the studied cohorts.

The bioinformatic analyses presented here represent the first broad investigation into specific, novel variants detected in lung cancer. While prior lung adenocarcinoma studies^24^,^25^ did investigate a set of lung cancer tissues but did not identify the mutations described here, it is important to stress that the present study involved a different cohort of patients with potentially different characteristics and exposures, and that none of these mutations occurred in more than one sample. As such, they should still be treated as isolated events until and unless they are found in additional lung cancer patient samples. One important question that remains from these analyses is whether the novel mutations described here are somatic or germline in nature, as this information may provide some insight into the genetic background underlying lung cancer. Because these are isolated mutations that only occurred in one specimen each, and because of the nature of lung cancer and its relationship to environmental and tobacco exposure, it is almost certain that these mutations are somatic; however, their status is unknown at this time.

It is notable that the G723S and A742V mutations have been described at analogous sites within other kinase domains in a variety of different diseases. A similar investigation was undertaken for a conserved residue pair in *EGFR*^29^. Importantly, both of the mammalian variations analogous to G723S in *CDC42BPG*, a downstream effector of Cdc42 capable of participating in cytoskeletal reorganization, and *STK38L*, a serine/threonine kinase important in neuronal cell differentiation, were detected in lung adenocarcinoma, providing an important contextual precedent for the potential role the G723S may have in contributing to the development of lung cancer. That these two proteins have overlapping functions and pathways with the known signaling pathways and cellular functions that EPHB4 participates in only contributes to the precedent.

The A742V mutation also has several cancer-associated precursors, one of which was also found in lung adenocarcinoma. Notably, the *EGFR* A839T variant has been associated with response to gefitinib in lung cancer^30^, and the A876V variant of *RET* occurring in medullary thyroid carcinoma was found to lie within its catalytic loop, suggesting a possible aberration in kinase activity^31^,^32^. The A742 residue is also known to lie within the catalytic loop of EPHB4^33^.

The degree to which these NS *EPHB4* mutations occur exclusively from aberrations in common cancer-associated genes is remarkable. One emerging philosophy in the study and treatment of cancer, especially lung cancer, is the phenomenon of driver and passenger mutations, whereby multiple mutations in a given tumor may be present, but only one or a few of these act to induce hyperproliferation and tumor progression —”driver” mutations—while others may simply be present as a result of prolonged genomic instability, carcinogen exposure, or other factors contributing to mutagenesis but not necessarily contribute to the malignant drive of a tumor cell—a “passenger” mutation^34^,^35^,^36^. Additionally, mutations commonly arise in tumors either from direct exposure to or as an evolutionary consequence of treatment with chemotherapeutic agents, raising the possibility of *EPHB4* mutations occurring in our cohort as a mechanism of resistance to therapy. Some of the most common genetic aberrations in lung cancers are mutations in *TP53*, *PIK3CA*, *PTEN*, *KRAS*, and *EGFR*. In light of these circumstances, it is remarkable that of six *EPHB4* mutation-harboring tissues, four had only one mutation among this broad set of cancer genes (R248Q and G245C in *TP53*, H1047R in *PIK3CA*, and G12C in *KRAS*), and two were wild-type at every site investigated (one of these two harbored the P881S mutation). These findings suggest that these *EPHB4* mutations occur in the absence of many other common lung cancer aberrations and that they therefore may be acting as driver mutations, especially in those cases in which no other mutations could be identified. A number of loci statuses were not determined, which makes a definitive conclusion more difficult, but these data do suggest an interesting trend.

Mutations in *EPHB4* have not been previously linked to cellular behavior, and these data provide the first evidence that *EPHB4* mutations in lung cancer may confer growth advantages as well as differences in protein modifications. In particular, two of the mutations resulted in decreased phosphorylation, a state typically associated with deactivation of kinase activity. However, several prior studies have demonstrated ephrin-independent activity of EPH receptors^37^,^38^,^39^,^40^. Constitutive kinase activation in EPHB4 harboring mutations is one possibility that may explain this. Though these mutants appear to bind ephrin-B2 ligand, they do not require ligand stimulation to effect changes in cellular growth and behavior. Ligand-independent signaling has been observed in many other RTKs and RTK mutants, including MET, RON, and EGFR. Future studies investigating the role of these variations in an *in vivo* setting are warranted given these promising findings in cultured cells. Furthermore, further characterization of these mutations from a structural and biochemical perspective would also provide crucial information toward understanding not only the nature of the variations themselves but also EPHB4 signaling generally.

It would also be interesting to test the effects of the remaining novel mutants detected in lung cancer. For example, the A230V mutation was associated with an extremely limited survival time (three months) and was also not found to co-occur with other cancer-associated variations, suggesting that it, too, may play a significant role in lung cancer. In contrast, the A371V and P381S mutations, which were detected in the same patient and which co-occurred with a codon 12 mutation in *KRAS*, were associated with a survival time approaching 10 years. These survival relationships may simply reflect the stage of disease; however, while the P381S mutant was predicted using CanPredict to likely be associated with cancer, the A371V variant was not. Given that it co-occurs, then, with a known activating mutation in *KRAS* and another predicted in *EPHB4*, it is possible that the A371V variant acts to protect against tumorigenesis.

The PamGene platform has been used frequently and in various iterations to demonstrate gene expression^41^, biochemical mechanisms^42^, rational personalized therapy selection and design^43^,^44^,^45^, drug target discovery^46^,^47^, and microbe species detection and identification^48^,^49^. Many of these studies have involved cancer signaling, treatment, and target identification and characterization. Among fellow RTKs, EPHA1, EPHA2, FGFR2, PDGFRß, Ret, and VEGFR2 have often been linked to cancer. In particular, EPHA2 has a demonstrated role in NSCLC^1^,^50^,^51^, breast cancer^52^, ovarian cancer, and brain cancer, among others. Expression of mutant EPHB4 resulted in increased EPHA2 phosphorylation. Based on our *in vitro* proliferation data indicating that these mutations confer a gain of function with respect to EPHB4, these data may therefore suggest that mutant EPHB4 forms potentially interact somehow, either directly or indirectly, with EPHA2, resulting in its enhanced phosphorylation. However, one interesting report suggested that EPHA2 activity may be less dependent on tyrosine phosphorylation than serine phosphorylation. EPHA2 kinase activity and pro-tumor effects were found to be correlated with Ser897 phosphorylation mediated by Akt; interestingly, this was a ligand-independent event, whereas binding of ephrin-A1 ligand was sufficient to abrogate EPHA2 Ser897 phosphorylation and therefore EPHA2 kinase activity^53^. Therefore, it would be interesting to also examine the interplay between EPHB4 modulation and EPHA2 serine phosphorylation, potentially through an Akt mediator. We also noted that the EPHB4-A742V variant was able to effect broad signaling changes without apparent phosphorylation following ephrin-B2 stimulation. It may be the case that, for this particular variation, dimerization is critical for phosphorylation and activation. Additionally, the assays used here addresses tyrosine phosphorylation but does not account for sites of serine/threonine phosphorylation, which may be important for effecting the changes in growth and behavior that we observed in this case. If EPHB4 mutants cause increased Akt signaling activity, then this may provide some understanding of the overlapping roles of EPHB4 and EPHA2. Expression of PDGFRß and Ret also increased with EPHB4 knockdown, and VEGFR2 phosphorylation increased with expression of EPHB4 mutants. As with EPHA2, it is possible that mutant forms of EPHB4 interact somehow with these receptors or effectors of their regulation to promote increased phosphorylation. In addition, CDK2 also had increased phosphorylation with mutant EPHB4 expression, indicating that these variants may be sufficient for promoting the cell cycle. Interestingly, CDK2 phosphorylation and activation of cell cycle progression has also been shown to be mediated by Akt^54^. This underscores the need to further explore the relationship between EPHB4 signaling and Akt activation, and it would be interesting to explore the efficacy of dual EPHB4 and Akt inhibition.

The most apparent and perhaps surprising finding to come out of these assays was the differential regulation of the STAT family of molecules. STATs interact with RTKs and other membrane-bound receptors; upon receptor stimulation, STATs can dimerize, become phosphorylated, and translocate to the nucleus to activate transcription of survival and anti-apoptotic genes such as Myc, XIAP, Bcl-XL, Bcl-2, survivin, and Fos^55^,^56^,^57^. While they are classically associated with cytokine signaling, STATs have long been known to play a role in the signaling of cancer cells as well. STAT5A was recently shown to be overexpressed, along with EPHA2, in HNSCC, and this signaling pair was found to positively predict response to chemoradiation therapy; STAT1 and STAT3 were overexpressed as well^58^. STAT3 was also shown to be phosphorylated after exposure to EGF^59^, and some mutations in the *FLT3* RTK have also been reported to activate STAT3 and STAT5^60^. Another study found that a form of *EPHB4* that was mutated at a site analogous to a common activating mutation in *MET*, *KIT*, and *RON* was sufficient to induce STAT3 phosphorylation^33^. Interestingly, STAT6 and STAT1 seem to play opposite roles in Hodgkin lymphoma; upon STAT6 knockdown, STAT1 was found to be upregulated in response^61^. Therefore, these previous studies have shown that multiple STATs are expressed and phosphorylated in human cancers, that they interact with RTKs and other receptors, and that they may be reciprocally regulated with one another in response to cellular stresses.

The data presented here demonstrated that while STAT6 phosphorylation increases with re-expression of wild-type EPHB4, indicating a pro-survival response, STAT5A phosphorylation was decreased with EPHB4 knockdown. It may be the case that STAT5A acts as an attempted survival mechanism upon knockdown of EPHB4 and the resulting lack of growth stimulation and that this role is distinct from STAT6 in this cell line. Relative to wild-type EPHB4, two point mutations in EPHB4 caused decreased phosphorylation of STAT3, and the P881S mutation decreased the phosphorylation of STAT5A and STAT6. It is therefore possible that these mutations activate other survival pathways outside of the STAT family or that other STATs are incrementally and concurrently phosphorylated below the threshold of detection in this assay as an alternate proliferation signal in the presence of these activating mutations.

A summary overview of the present findings is shown in Table 3. As with any broad systems-based approach, these studies have provided valuable information but have also raised many novel and sequential questions in the process. Further studies into the nature of these relationships are warranted to elucidate the full scope of EPHB4 biology in lung cancer cells.

## Additional Information

**How to cite this article**: Ferguson, B. D. *et al.* Novel *EPHB4* Receptor Tyrosine Kinase Mutations and Kinomic Pathway Analysis in Lung Cancer. *Sci. Rep.* **5**, 10641; doi: 10.1038/srep10641 (2015).

## Supplementary Material

Supplementary Information

## Figures and Tables

**Table 1:**
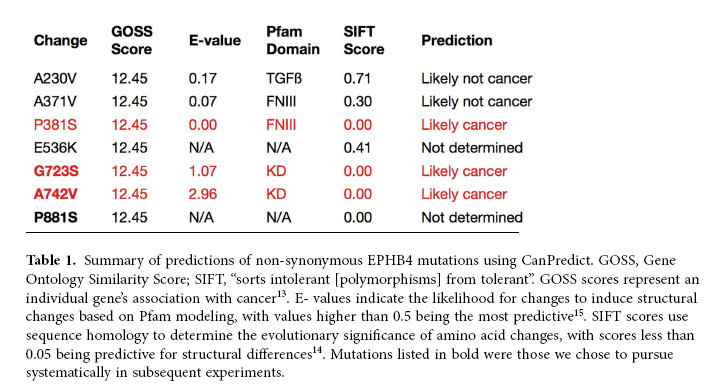
Summary of predictions of non-synonymous EPHB4 mutations using CanPredict.

**Table 2:**
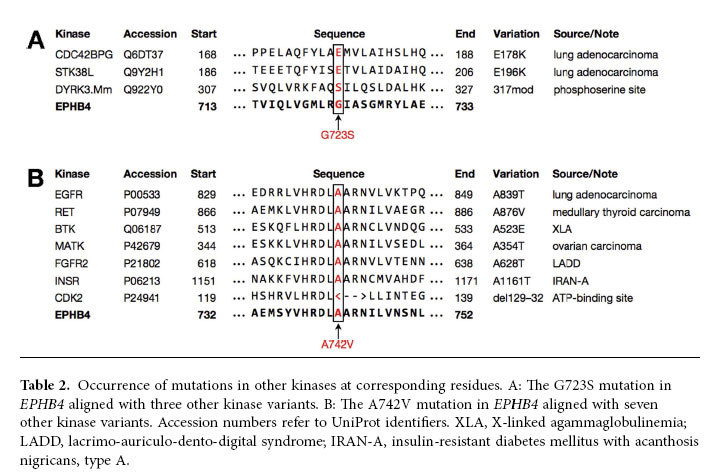
Occurrence of mutations in other kinases at corresponding residues.

**Table 3:** Summary overview of findings.

	**G723S**	**A742V**	**P881S**
**PamGene**	increased phosphorylation of CDK2, EPHA2, EPOR decreased phosphorylation of STAT3	increased phosphorylation of CDK2, EPHA1, EPHA2, EPOR, FGFR2, PDGFRß, PECAM1, RET, VEGFR2 decreased phosphorylation of STAT3	increased phosphorylation of CDK2, EPHA1, EPHA2, EPOR, FGFR2, PDGFRß, PECAM1, RET, VEGFR2 decreased phosphorylation of STAT5A, STAT6
**CanPredict**	likely associated with cancer SIFT score = 0.00	likely associated with cancer SIFT score = 0.00	SIFT score = 0.00
**mCluster**	analogous mutations in CDC42BPG in human lung adenocarcinoma, STK38L in human lung adenocarcinoma, DYRK3 (*M. musculus* phosphoserine site)	analogous mutations in EGFR in human lung adenocarcinoma, RET in human medullary thyroid carcinoma, BTK in human XLA, MATK in human ovarian carcinoma, FGFR2 in human LADD, INSR in human IRAN-A, CDK2 (human ATP-binding site)	no established analogous mutations
**Structure + Function**	creates potential phosphoserine site	potentially disrupts adjacent L741 residue from the short helix structure involving R739, D740, and L741	creates potential phosphoserine site potentially disrupts turn within secondary structure

Novel EPHB4 mutants investigated here are listed with findings compiled from bioinformatic analyses and a systems-based approach.
