# Supplementary material for: Novel EPHB4 Receptor Tyrosine Kinase Mutations and Kinomic Pathway Analysis in Lung Cancer
Source: Sci Rep. 2015 Jun 15;5:10641. doi: 10.1038/srep10641 (PMC4466581; doi:10.1038/srep10641)

**Novel *EPHB4* receptor tyrosine kinase mutations and kinomic pathway analysis in lung cancer**

Benjamin D. Ferguson, Yi-Hung Carol Tan, Rajani S. Kanteti, Ren Liu, Matthew J. Gayed, Everett E. Vokes, Mark K. Ferguson, A. John Iafrate, Parkash S. Gill, Ravi Salgia

**Supplementary Figure and Table Legends**

*Supplementary Figure 1. Expression of EPHB4 in a non-EPHB4-expressing lung cancer cell line.*

H661 was transfected with wild-type EPHB4 or EPHB4 harboring one of three mutations identified in lung cancer tissues. Untransfected cells and cells treated with transfection reagent only served as negative controls.

*Supplementary Figure 2. Exogenous EPHB4 expression in 293T cells.*

Immunofluorescence was performed using anti-EPHB4 primary antibody (#131). Expression of EPHB4 was localized to the cellular membrane.

*Supplementary Figure 3. Chromatograms demonstrating eight non-synonymous* EPHB4 *mutations detected in lung tumor tissues.*

Forward (top half of each panel) and reverse (bottom half) sequencing reactions are shown.

*Supplementary Figure 4. Ephrin-B2 binding and phosphorylation among wild-type and mutant EPHB4.*

A: Ephrin-B2 binding as measured by optical density following incubation of whole cells expressing EPHB4 variants with ephrin-B2-AP followed by treatment with PNPP. B: Protein phosphorylation of EPHB4 variants displayed as the ratio of phosphorylated protein to total protein measured by immunoblot band intensity.

*Supplementary Figure 5. Expression of the EPHB4 A742V mutation enhances cellular motility.*

Cellular motility was assessed using standard wound healing assays using H661 cells following transfection of wild-type or mutant EPHB4. Time elapsed in hours is shown at left.

*Supplementary Figure 6. Validation of PamGene select tyrosine kinome data.*

Several representative peptide targets of EphB4 and its mutants that demonstrated significantly modulated phosphorylation patterns in the tyrosine kinome analysis were validated via immunoblotting. EV, empty vector. WT, wild-type EphB4. Individual EphB4 mutant constructs are additionally listed. ß-actin was used as a loading control for PVDF (top) and nitrocellulose (bottom) membranes.

*Supplementary Figure 7. Signaling network assembled by significantly altered kinases following* EPHB4 *knockdown.*

Cellular localization flows from left (external) to right (internal). Fold-change indicators are depicted by circles above and to the right of individual objects. In this case, red tones indicate increased phosphorylation and blue tones indicate decreased phosphorylation as detected using the PamGene platform.

*Supplementary Figure 8. GeneGo process network for anti-apoptosis mediated by external signals via MAPK and JAK/STAT.*

Highlighted are regulators (yellow) and targets (aqua) of STAT5A and STAT6 signaling.

*Supplementary Table 1. Primers used in exon sequencing, quantitative PCR, and site-directed mutagenesis.*

*Supplementary Table 2. Summary of* EPHB4 *variants detected in lung cancer tissue samples.*

The number of tested samples within each subtype is denoted in parentheses. "Unique" refers to the absolute number of variant sites identified; "novel" variants are denoted using a middle dot (•) and have not been identified previously in the literature or common SNP databases. ∆, base change; ∆AA, amino acid residue change.

*Supplementary Table 3. Detailed list of* EPHB4 *variations in lung cancer.*

Non- synonymous mutations appear in bold. Novel mutations not previously described are noted by a middle dot (•). EC, extracellular.

*Supplementary Table 4. Detailed summary of patient characteristics in patients harboring non-synonymous* EPHB4 *variants.*

Where available, variations (or the lack thereof) in other commonly aberrant proteins in lung cancer are noted. WT, wild-type; ND, not determined.

Supplementary Figure 1.


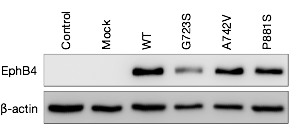


Supplementary Figure 2.


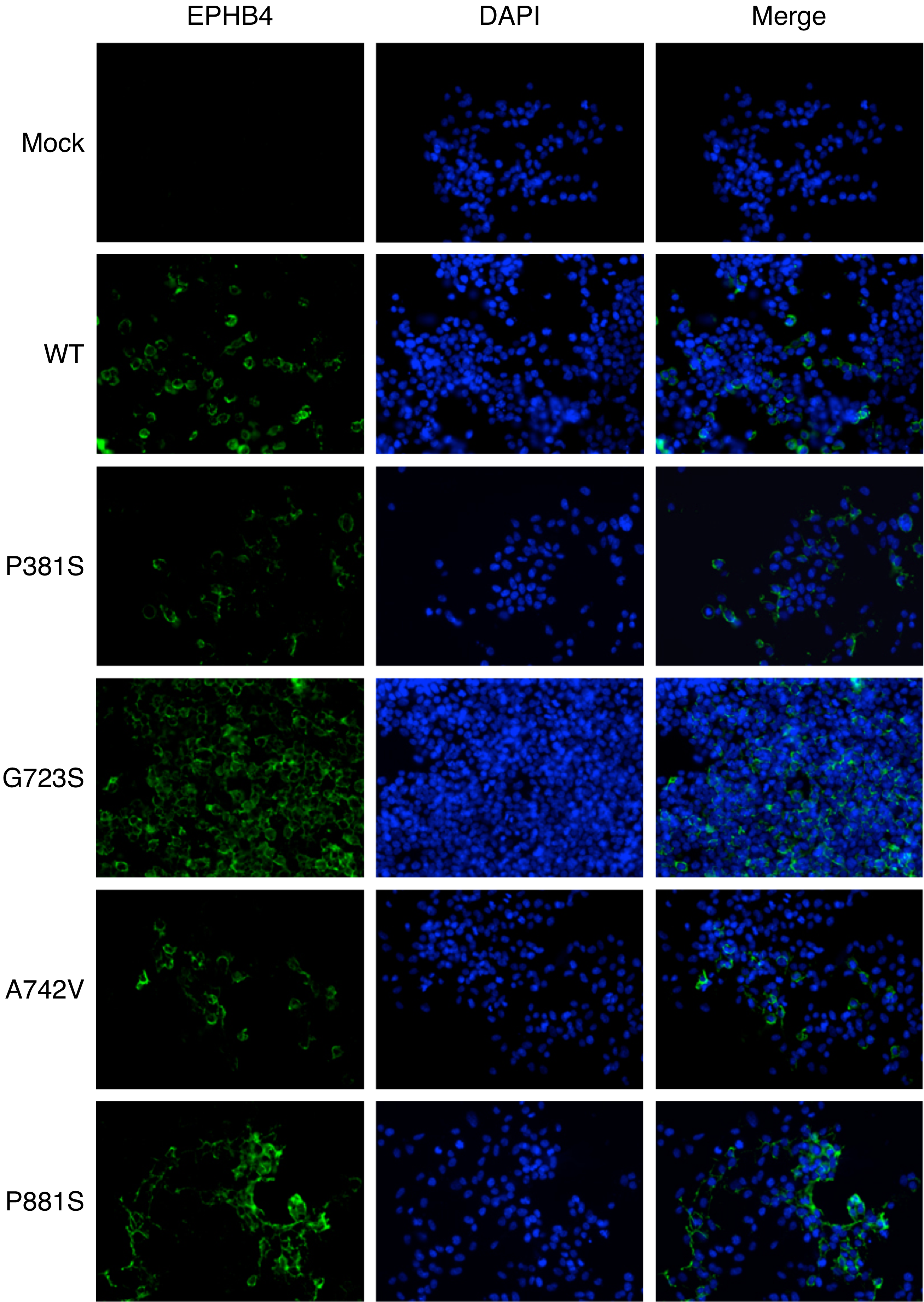


Supplementary Figure 3.


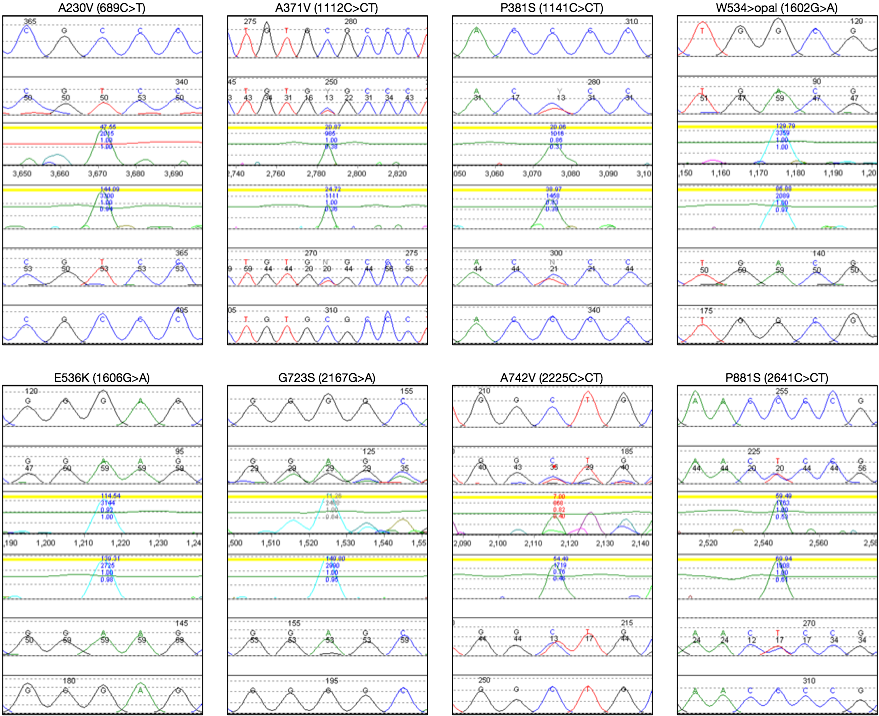


Supplementary Figure 4.


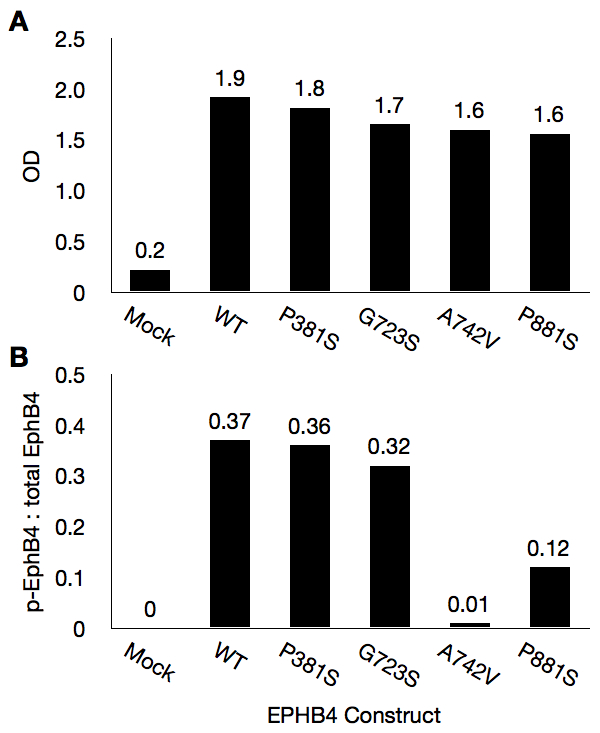


Supplementary Figure 5.


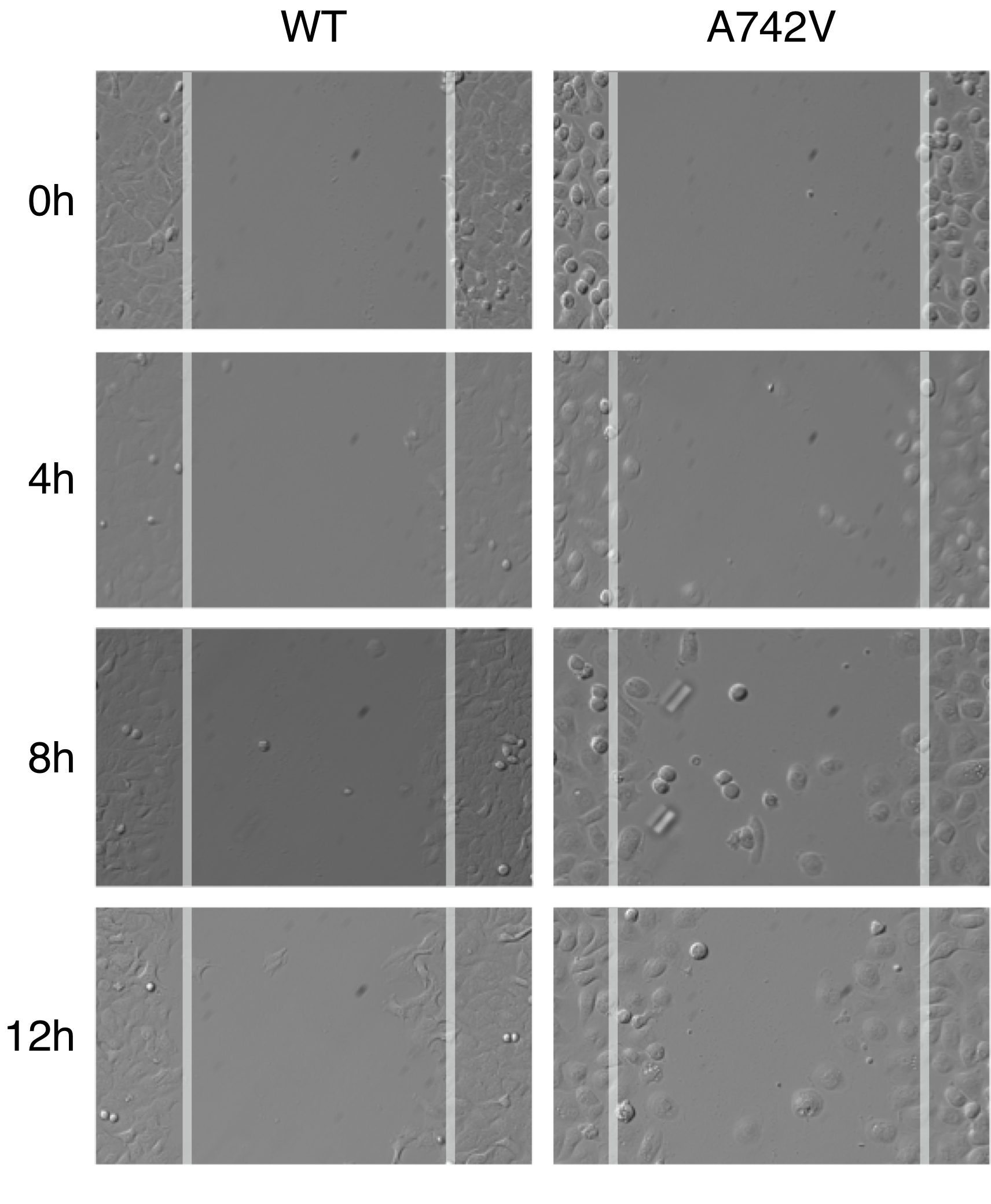


Supplementary Figure 6.


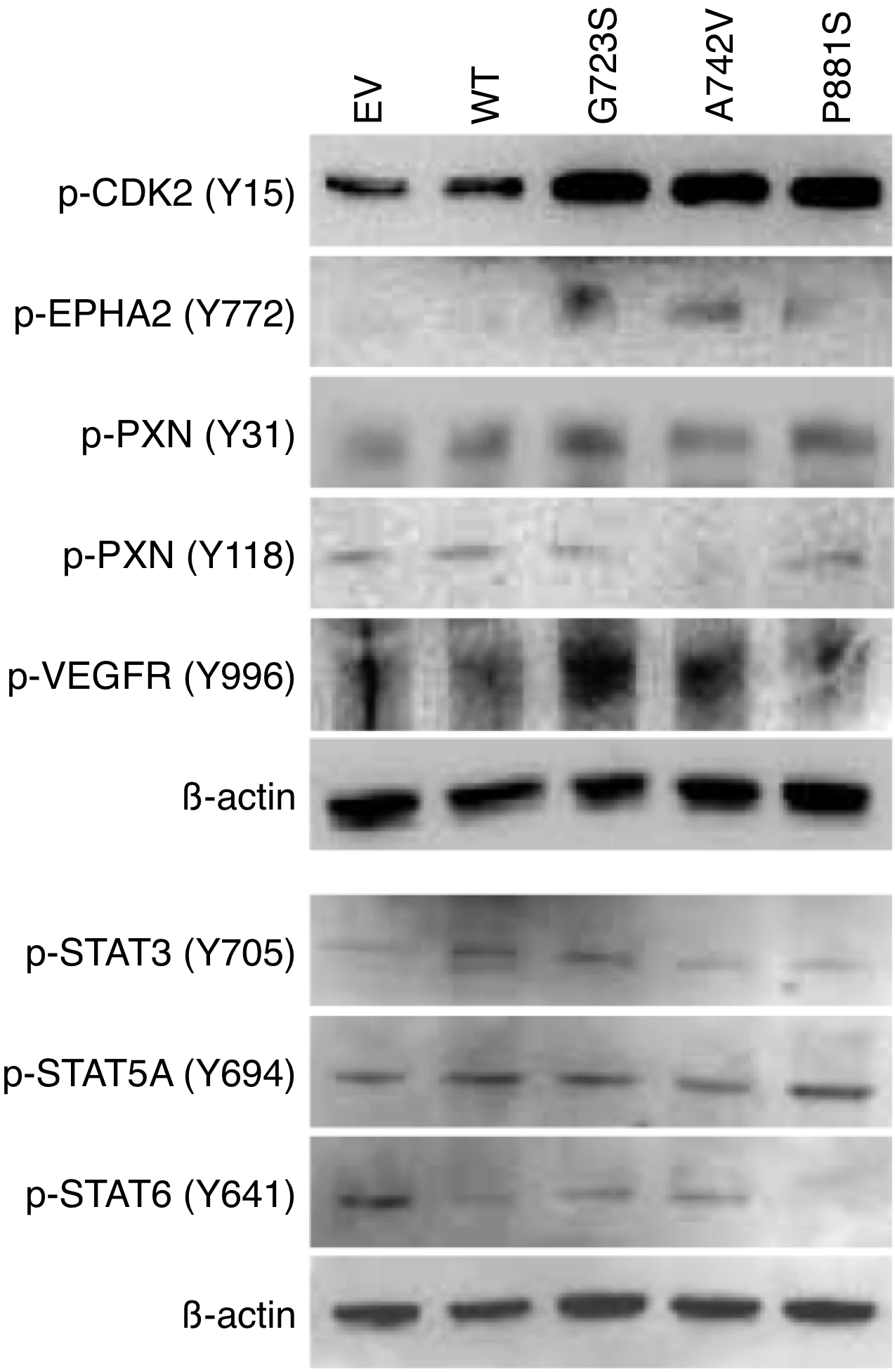


Supplementary Figure 7.


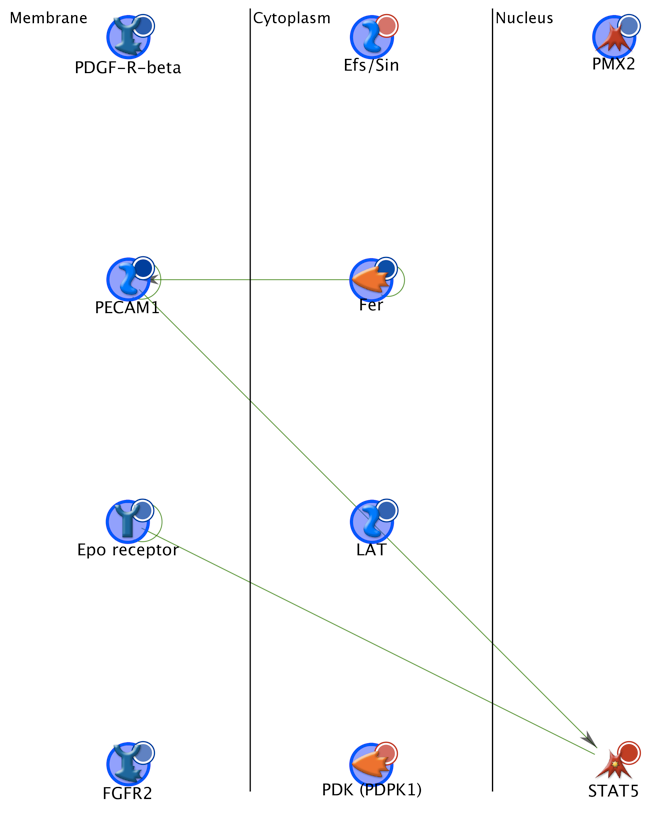


Supplementary Figure 8.


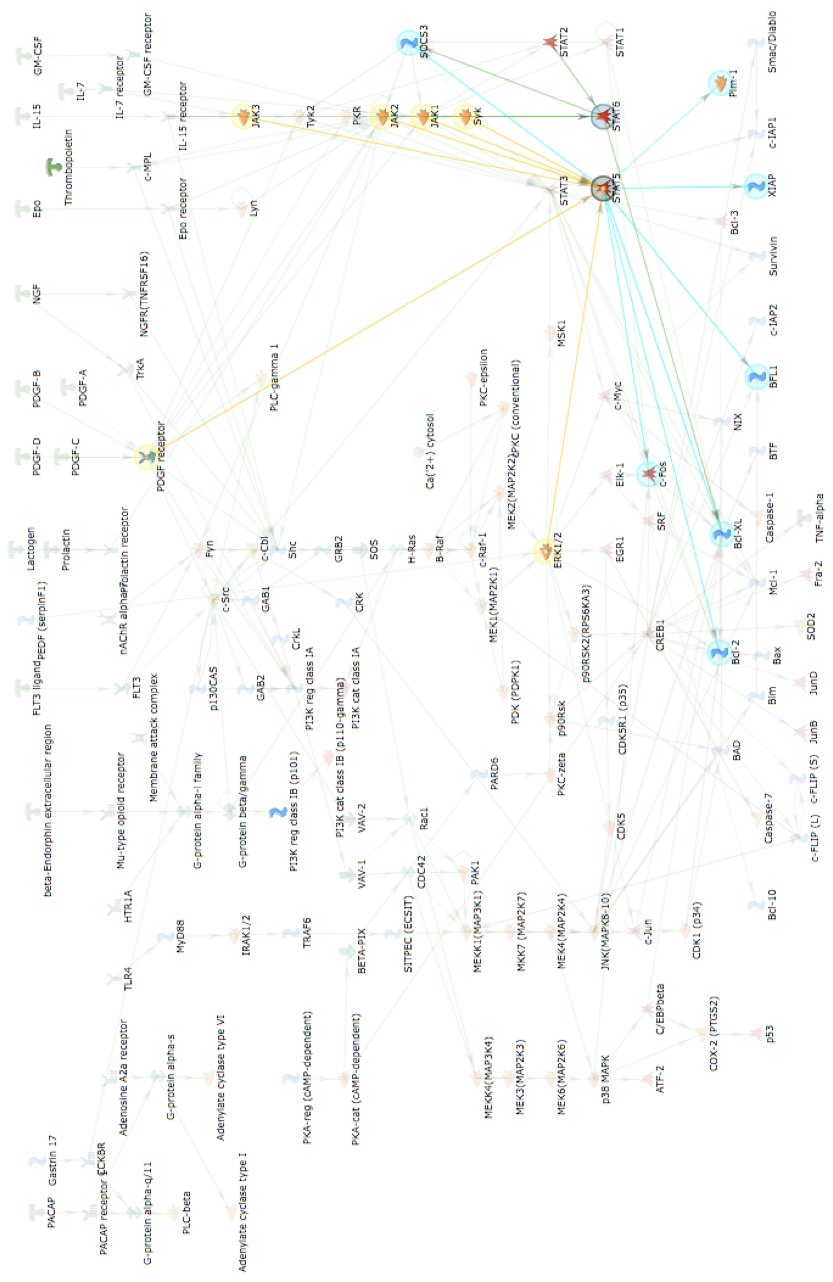


Supplementary Table 1.


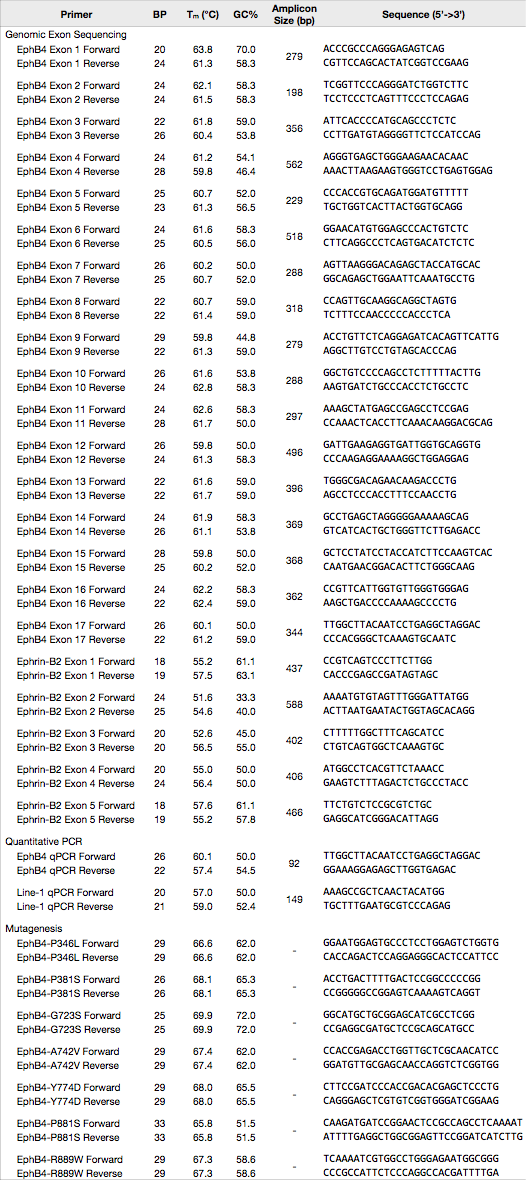


Supplementary Table 2.


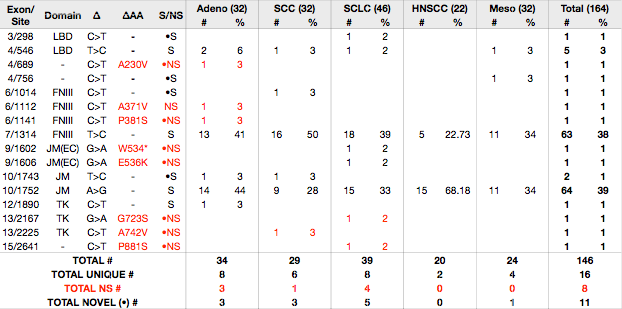


Supplementary Table 3.


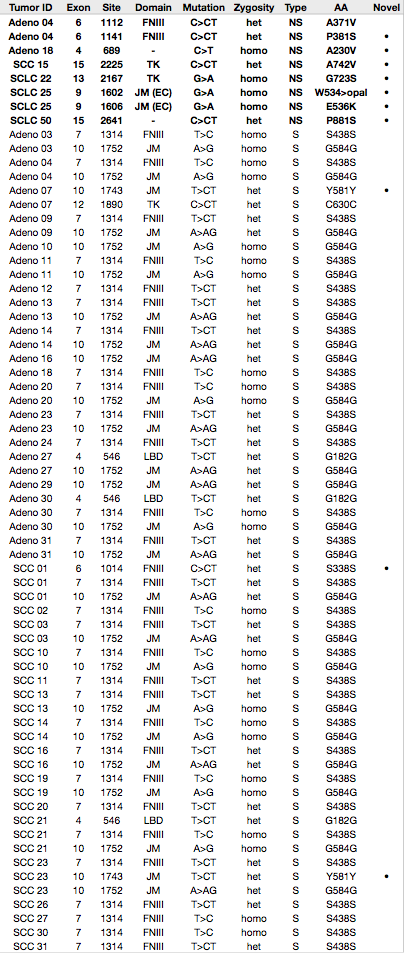

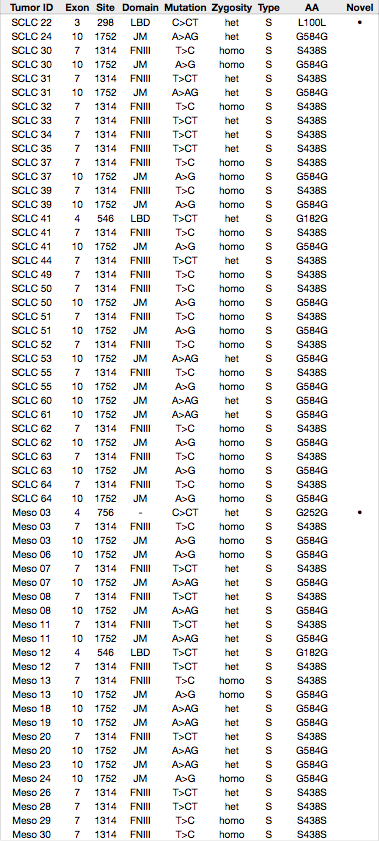


Supplementary Table 4.


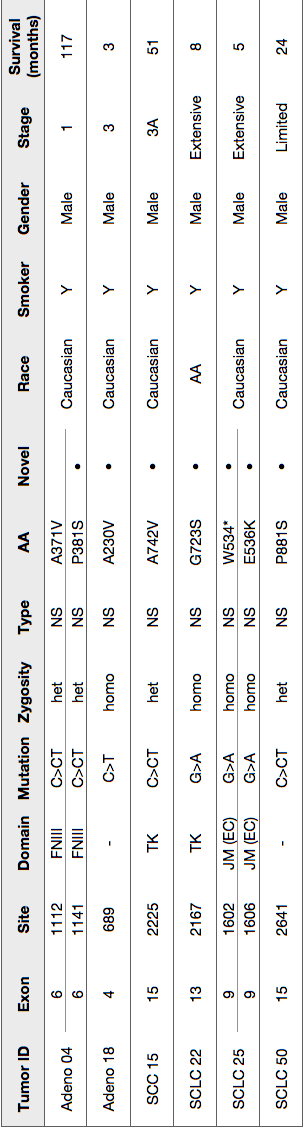

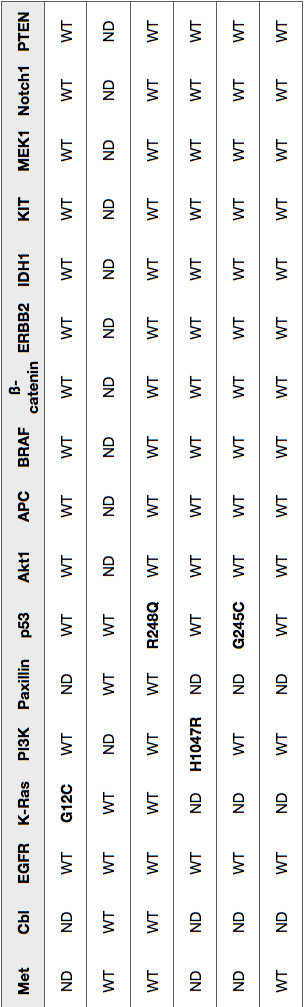

Supplement: Supplementary Information [file srep10641-s1.doc]
